# Understanding Societal Resilience—Cross-Sectional Study in Eight Countries

**DOI:** 10.3389/fpubh.2022.883281

**Published:** 2022-04-01

**Authors:** Moran Bodas, Kobi Peleg, Nathan Stolero, Bruria Adini

**Affiliations:** ^1^Department of Emergency and Disaster Management, Faculty of Medicine, School of Public Health, Tel-Aviv University, Tel Aviv-Yafo, Israel; ^2^National Center for Trauma and Emergency Medicine Research, Sheba Medical Center, The Gertner Institute for Epidemiology and Health Policy Research, Ramat Gan, Israel

**Keywords:** societal resilience, preparedness, contextual factors, target factors, multinational study

## Abstract

Civilian populations that are more prepared for emergencies are more resilient. Ample research has been carried out over the last three decades to identify the factors that contribute to public readiness to emergencies and disasters and enhance societal resilience. However, the analysis did not achieve an in-depth comprehension of the types of contributing factors, namely, contextual vs. target aspects. A cross-sectional study that explored attitudinal factors among civilian populations took place during the months of January–February 2021. Diverse representative samples (*N* ≥ 500 each) of adults from eight countries (Italy, Romania, Spain, France, Sweden, Norway, Israel, and Japan) were engaged. The primary outcomes of this study were individual and societal resilience as well as emergency preparedness. The results suggest that in most countries, levels of trust are relatively high for emergency services and health services, and relatively low for politicians. In the overall sample, the individual preparedness index, which delineates the compliance with general household adjustment recommendation for emergencies, averaged at 4.44 ± 2.05SD (out of 8). Some variability was observed between countries, with some countries (e.g., Spain, Norway, and Italy) reporting higher preparedness rates than others (e.g., Japan). In the overall sample, levels of individual resilience were mediocre. Multivariate analysis showed that the following variables are predictors of societal resilience: trust (β = 0.59), social norms and communality (β = 0.20), individual resilience (β = 0.05), individual preparedness (β = 0.04), risk awareness (β = 0.04), and age (β = 0.03). The results of this study show that there are commonalities and differences between societies across Europe and beyond concerning societal resilience at large, including preparedness, individual resilience, and risk perception. Despite socio-cultural driven differences, this study shows that societies share varied characteristics that may contribute toward a common model for assessing societal resilience and for explaining and predicting resilience and readiness.

## Introduction

Emergencies and disasters are detrimental to human lives and economies. According to the Research Center for the Epidemiology of Disasters (CRED), more than 7,300 disasters were recorded over the past 20 years, killing nearly 1.23 million people and affecting 4 billion more. These events caused an economic loss of close to US$ 2.97 trillion worldwide (1).

There is a consensus among scholars that civilian populations that are more prepared for emergencies are more capable of better reacting during the materialization of varied adversities, making them more resilient (2, 3). In contrast to national resilience, which deals with national infrastructure capacities to withstand and cope with hardships, societal resilience represents the ability of the members of the public to continue to function despite adversities.

According to the United Nations Office for Disaster Risk Reduction (UNDRR, formerly UNISDR), resilience is defined as follows: “In the context of disaster risk, the ability of a system, community or society exposed to hazards to resist, absorb, accommodate, adapt to, transform and recover from the effects of a hazard in a timely and efficient manner, including the preservation and restoration of its essential basic structures and functions through risk management” (4).

**Figure 1:**
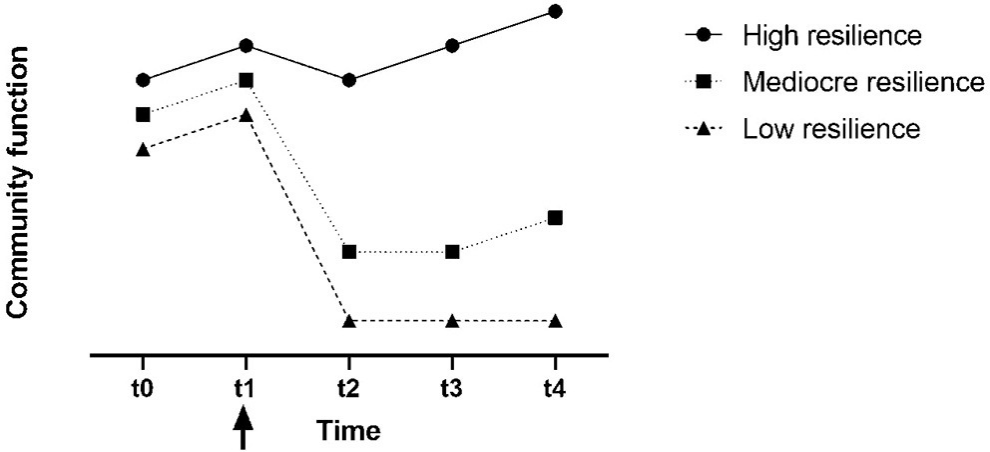
A hypothetical trajectory of community function based on their level of resilience. Arrow indicates the onset of crisis/disaster situation [Modified from Zhang (8) and Mayunga (9)].

The term resilience is derived from the Latin root “resiliere”, which means “to jump back”. Therefore, resilience is often referred to as the ability of a system (e.g., a society) to “bounce back” in face of adversity (5, 6). Societal resilience refers to “the capacity of communities to flexibly contain major disruptions and to rapidly bounce back and forward following the unavoidable decline of their core functionalities” [(7), p. 301]. Alternatively put, a resilient society is one that is able to absorb shocks caused by disasters, emergencies and crises, and recuperate so that the community returns to normal function rapidly and continues on a trajectory of growth. A social system with high resilience should be able to adapt and adjust itself without suffering a significant and long-lasting decline in its crucial functions while undergoing a crisis [see Figure 1; (10, 11)]. Resilience is dependent on flexibility and the capacity to dynamically adapt to changing conditions, considering the varied needs of relevant networks, time constraints and impact of internal and external stakeholders (12).

Resilience is proposed as the result of dynamic processes involving networks and nets, time demands, and the influences of external actors.

Scholars agree that one of the building blocks for societal capacity to cope in the face of hardship caused by disasters is the household. It is widely accepted that households engaging in preparedness activities are more resilient, due to both increased awareness and actual adjustments that contribute to the survivability of family members in the aftermath of disaster (13–18). Yet, despite this, levels of households preparedness reported in the literature continue to be insufficiently low for many populations (19). One of the primary reasons for this gap can be explained by the limited knowledge about the socio-psychological elements involved in the constructs of resilience and preparedness behavior (20).

Most recently, a 2019 publication by Douglas Paton summarizes the results of the vast and ongoing search for the determinants of societal resilience (21). The author demonstrated the multitude of aspects influencing preparedness and resilience on the individual, family, community, and society levels. The classification of the varied elements that may impact societal resilience can be broken down into two manageable categories—contextual vs. target factors. This is based on the assumption that the analysis needs to consider both fundamental (contextual) aspects that characterize each society and elements or actions (target aspects) that may mitigate or exacerbate disruptions and the response to them (22).

Contextual factors are elements that are inherent in society and are difficult to modify. Nevertheless, they can have a dramatic effect on determining the level of societal resilience. Examples of contextual factors include the following: (a) Socioeconomic status—Directly linked to vulnerability. Higher income is usually associated with higher resilience, presumably because higher income is also associated with better living conditions and more available resources to invest in preparedness (23). Although an individual feature, socioeconomic status can be considered a macro-economic component requiring state-driven policies to be changed, therefore it is considered a contextual factor; (b) Religiosity (affiliation to religion)—Being more religious is positively associated with resilience (24); Nevertheless, in some contexts, religiosity can lead to passiveness in action taking; (c) Family status, core family size, number of children under age 18; being in a relationship and especially having children, were found to be associated with resilience, presumably due to the existence of social bonds that are important for individual sense of belonging and self-efficacy (25); (d) Level of education—higher levels of education are associated with higher resilience (25); (e) Experience with risks (14, 26); (f) Personality traits (e.g., emotional stability, depression/anxiety, optimism, fatalism…)—optimistic people are more resilient than people that tend to be depressed or anxious (27); (g) Coping style—Rational thinking is more associated with preparedness (27); (h) Communality in place of residence—the greater the social network in a certain community, the greater the chances of that society to be resilient in face of adversity (28–30); and (i) Social norms in place of residence (21).

In contrast to contextual factors, target factors are individual and societal characteristics that can be more easily modified, therefore placing them as prime candidates for intervention plans to promote societal resilience. Examples of target factors include the following: (a) Risk awareness—being more aware of risks is likely to be associated with increased resilience and readiness (31); (b) Threat perception (perception of likelihood, severity, threat intrusiveness)—increased perception of likelihood, severity or threat intrusiveness are likely to increase readiness, and therefore resilience (19); (c) Perception of responsibility—specifically, the tendency to either assume preparedness responsibility is associated with higher preparedness and resilience (32); (d) Perception of response (efficacy, cost-effectiveness, self-efficacy to perform, etc.) (33); (e) Coping skills (34); (f) Level of trust (in local and national entities) (35); (g) Sense of preparedness (19, 27); (h) Actual household/ neighborhood/ regional/ national preparedness (36); and (i) Beliefs, such as fatalism, optimism, etc. (21).

Engaging in a more in-depth analysis of resilience determinants and better understanding the premises of resilience are of crucial importance for the advancement of policies, interventions, and actions. This is especially required to achieve sustainable development of societies that can face future challenges, adapt and adjust to their expected hardships, and recover more quickly from disasters.

This study aimed to identify and analyze contextual and target aspects amongst civil societies in varied countries (6 within and 2 beyond the EU). The study focused on the perceptions and beliefs of the public in each studied society, as it was previously found that willingness of individuals to contribute toward emergency preparedness is dependent on their perceived assessment of the fairness of the institutionalized authorities and responders (37). Specifically, this study, conducted in the framework of the ENGAGE Project funded by the European Commission was to (1) compare the perceptions of diverse populations concerning aspects associated with social resilience, to identify commonalities and diversities among diverse national and local groups; (2) to understand the relationships between risk awareness and actual resilience among different civil societies; and (3) to map the trust of citizens in varied responders and authorities to understand its impact on their societal resilience.

This study sought to explore factors associated with societal resilience and risk awareness. Therefore, the main research question for this study was “what are the contextual and target factors that are associated with societal resilience?” We hypothesized that higher reported levels of perceived communality, coping skills, trust in authorities, and perception of personal responsibility will be associated with higher levels of individual and societal resilience and preparedness. The study aims to solidify further our understanding of societal resilience in the context of the theoretical framework presented above by suggesting which factors are predictive of societal resilience.

## Materials and Methods

### Study Type, Population, and Sampling

This is a cross-sectional study that explored attitudinal factors as expressed by diverse samples of target countries across Europe, Israel, and Japan. The study took place during the months of January–February 2021.

This study included participants from eight countries: Italy, Romania, Spain, France, Sweden, Norway, Israel, and Japan. In each country, the target population was the adult population of the country (>18 years). According to the calculation of “proportion” sample size, based on OpenEpi, in each country, there would need to be 385 respondents in each society (38). To maintain working frameworks and consistency across studied countries, a national, diverse, and representative sample of at least 500 participants was obtained in each country. The internet panel company used the stratified sampling method, based on data published by the Central Bureau of Statistics from each country concerning age, gender, and geographic locations. The countries were chosen to reflect varied populations' characteristics, including from Western and Eastern European countries, as well as two countries beyond the EU (Japan and Israel).

### Tools and Variables

The online questionnaire used for this study was based on several validated tools, as well as tools developed specifically for this study. The final questionnaire included 75 items pertaining to different constructs as described in the following.

#### Primary Outcomes (Dependent Variables)

The primary outcomes assessed in this study included the following:

**Individual resilience**—this construct was assessed with a three items questionnaire on a Likert scale ranging between 1 (“Not true at all”) to 5 (“True nearly all the time”). The tool was based on the Connor-Davidson Resilience Short Scale, 2003—abbreviated version (39). The tool was used with the consent of the authors. The tool measures the perception of individual resilience. An example of an item in this scale is “I am able to adapt when changes occur”. Considering the aims of the study, one item was added; “I know the basic emergency rules that I should follow in case of an emergency”. The index has a Cronbach Alpha score of 0.821 and was generated by computing the mean of responses to all three items.

**Societal resilience**—this construct was assessed with an eight items questionnaire on a Likert scale ranging between 1 (“strongly disagree”) to 5 (“Strongly agree”). The tool was based on a validated tool developed by Kimhi et al. (35). The tool measures the perception of societal resilience. An example of an item in this scale is “I have full confidence in the ability of the emergency services of my country to protect our population”. The index has a Cronbach Alpha score of 0.905 and was generated by computing the mean of responses to all eight items.

**Actual emergency preparedness**—this construct was assessed with an eight items binary (yes/no) questionnaire. The tool seeks to explore which of the eight items needed for household preparedness are reported as complied-with by the participant. The tool was based on a validated tool by Bodas et al. (19). The index was generated by computing the sum of preparedness actions reported as complied with by the participants and ranged from 0 to 8. In addition, four more items were included to assess preparedness on the communal level, including the existence of public shelters, a network of assistance, good access to emergency services, and an emergency plan. The index was generated by computing the sum of preparedness actions reported as complied with by the participants and ranged from 0 to 4.

#### Secondary Outcomes (Independent Variables)

Secondary outcomes assessed in this study included the following:

**Social norms and sense of communality**—this construct was assessed with a six items questionnaire on a Likert scale ranging between 1 (“strongly disagree”) to 5 (“Strongly agree”). The tool was based on a validated tool by the authors (unpublished data). An example of an item in this scale is “There is mutual assistance and people care for one another”. The index has a Cronbach Alpha score of 0.880 and was generated by computing the mean of responses to all six items.

**Coping skills, styles, and resources**—this construct was assessed with a four items questionnaire on a Likert scale ranging between 1 (“Does not describe me at all”) to 5 (“Describes me very well”). The tool was based on a validated tool by Sinclair and Wallston (40). An example of an item in this scale is “Regardless of what happens to me, I believe I can control my reaction to it”. The index has a Cronbach Alpha score of 0.798 and was generated by computing the mean of responses to all four items.

**Perception of trust and responsibility**—these constructs were assessed with two 8-items questionnaires on a Likert scale ranging between 1 (“Not at all”) to 5 (“Very much”). In each questionnaire, participants were presented with a set of eight entities (e.g., government, local authority, first responders, media, etc.) and were asked to rank their levels of trust and responsibility to prepare for emergencies, respectively. The tools were based on a validated tool by Tsur et al. (41) and Kimhi et al. (35). The indices have a Cronbach Alpha score of 0.869 for trust and 0.861 for responsibility. They were generated by computing the mean of responses to all eight items, separately for trust and responsibility.

**Prior exposure to a major disaster**—this item was assessed with a single yes/no question: “Except for COVID-19, have you been personally exposed in the past 5 years to a significant disaster risk?”

**Socio-demographics**—the questionnaire also assessed socio-demographic variables, including gender, age, nationality, place of residence, familial status, number of children under the age of 18, affiliation to religion, level of religiosity, education, income, and a sense of belonging to a specific community.

### Study Data and Data Collection

Data acquisition was conducted through the service of *iPanel*, a public opinions polling service in Israel. Since 2006, the iPanel provides an online platform for a wide variety of information collection services, including polls and public opinion surveys. It adheres to the stringent standards of the world association for market, social, and opinion researchers (ESOMAR). iPanel was contracted to computerize the online questionnaire in all eight languages and to sub-contract local vendors in each country to facilitate the dissemination of the questionnaire in each participating country.

All data collected was obtained through responses provided by participants in each of the participating countries to an online anonymous questionnaire. Questionnaires were presented in eight languages: Spanish, Romanian, Swedish, Norwegian, Italian, Japanese, French, and Hebrew. Each language was used in its respective country. Validation of the translation process to each language was obtained through reverse translation into English and comparison to the original version of the tool. Data was collected into spreadsheets and was collated into a single database on which statistical analysis was conducted.

### Statistical Analysis

Statistical analysis was conducted using SPSS (ver. 27). The analysis included both descriptive and analytical methods. Before analysis, indices were generated and their reliability was assessed using Cronbach's Alpha. The Chi-square test was used to evaluate the difference in proportions of variables between groups. Independent samples *t*-test or Mann-Whitney's U test were used to compare means between independent samples. Spearman R test was used to assess the correlation between continuous variables. Two separate multivariate linear regression analyses were used to predict the two primary outcomes (dependent variables) reported resilience and emergency preparedness. Only variables found to be associated with the dependent variables in the univariate analysis were introduced into the analyses. Regression performed in Enter mode. In all statistical analyses performed, a *p*-value of 0.05 or less was determined as statistically significant.

## Results

### Secondary Outcomes

#### Sample Characteristics

The overall sample of this study included 4,013 participants from eight countries: Israel, Sweden, Norway, Romania, Spain, France, Italy, and Japan. No statistical significances were observed between samples concerning the proportion of gender and the mean age. Table 1 provides the complete socio-demographic breakdown of the studied samples.

**Table 1:** Socio-demographic breakdown of the studied sample (*N* = 4,013).

**Variable**	**Israel** ***N* = 504**	**Sweden** ***N* = 504**	**Norway** ***N* = 500**	**Romania** ***N* = 500**	**Spain** ***N* = 502**	**France** ***N* = 503**	**Italy** ***N* = 500**	**Japan** ***N* = 500**
**Gender**								
Female	258 (51.1%)	247 (49.0%)	236 (47.2%)	253 (50.6%)	245 (48.8%)	247 (49.1%)	243 (48.6%)	245 (49.0%)
Male	246 (48.7%)	257 (51.0%)	264 (52.8%)	247 (49.4%)	257 (51.2%)	256 (50.9%)	257 (51.4%)	255 (51.0%)
**Age**								
Average ± SD	39.93 ± 14.10	39.84 ± 13.65	40.11 ± 13.65	38.76 ± 12.99	39.03 ± 12.60	40.16 ± 13.05	40.17 ± 12.72	39.97 ± 12.73
Up to 24 (“Gen Z”)	89 (17.7%)	78 (15.5%)	85 (17.0%)	84 (16.8%)	69 (13.7%)	64 (12.7%)	60 (12.0%)	67 (13.4%)
25-40 (“Millennials”)	179 (35.5%)	195 (38.7%)	168 (33.6%)	199 (39.8%)	220 (43.8%)	208 (41.4%)	206 (41.2%)	196 (39.2%)
41-56 (“Gen X”)	157 (31.2%)	165 (32.7%)	187 (37.4%)	158 (31.6%)	152 (30.3%)	163 (32.4%)	167 (33.4%)	169 (33.8%)
57 and above (“Boomers”)	79 (15.7%)	66 (13.1%)	60 (12.0%)	59 (11.8%)	61 (12.2%)	68 (13.5%)	67 (13.4%)	68 (13.6%)
**Religion**								
Christian - Protestant	0 (0.00%)	137 (27.2%)	142 (28.4%)	15 (3.0%)	12 (2.4%)	21 (4.2%)	9 (1.8%)	8 (1.6%)
Christian - Catholic	0 (0.00%)	39 (7.7%)	47 (9.4%)	37 (7.4%)	270 (53.8%)	202 (40.2%)	341 (68.2%)	10 (2.0%)
Christian - Other	0 (0.00%)	53 (10.5%)	74 (14.8%)	382 (76.4%)	20 (4.0%)	12 (2.4%)	10 (2.0%)	4 (0.8%)
Muslim	1 (0.2%)	33 (6.5%)	26 (5.2%)	4 (0.8%)	6 (1.2%)	23 (4.6%)	2 (0.4%)	2 (0.4%)
Jewish	491 (97.4%)	5 (1.0%)	2 (0.4%)	0 (0.0%)	2 (0.4%)	1 (0.2%)	0 (0.0%)	4 (0.8%)
Other	0 (0.00%)	18 (3.6%)	19 (3.8%)	18 (3.6%)	12 (2.4%)	17 (3.4%)	12 (2.4%)	130 (26.0%)
Atheist / No religion	12 (2.4%)	219 (43.5%)	190 (38.0%)	44 (8.8%)	179 (35.7%)	226 (44.9%)	126 (25.2%)	342 (68.4%)
**Religiosity**								
Highly religious	80 (15.9%)	62 (12.3%)	25 (5.0%)	33 (6.6%)	26 (5.2%)	29 (5.8%)	42 (8.4%)	21 (4.2%)
Religious	104 (20.6%)	157 (31.2%)	168 (33.6%)	309 (61.8%)	168 (33.5%)	132 (26.2%)	251 (50.2%)	76 (15.2%)
Not religious	320 (63.5%)	284 (56.3%)	307 (61.4%)	158 (31.6%)	307 (61.2%)	341 (67.8%)	207 (41.4%)	400 (80.0%)
**Family status**								
Coupled with children	285 (56.5%)	158 (31.3%)	150 (30.0%)	244 (48.8%)	244 (48.6%)	236 (46.9%)	223 (44.6%)	157 (31.4%)
Coupled w/o children	81 (16.1%)	152 (30.2%)	127 (25.4%)	69 (13.8%)	109 (21.7%)	110 (21.9%)	98 (19.6%)	65 (13.0%)
Single with children	36 (7.1%)	28 (5.6%)	48 (9.6%)	32 (6.4%)	28 (5.6%)	44 (8.7%)	20 (4.0%)	25 (5.0%)
Single w/o children	102 (20.2%)	166 (32.9%)	175 (35.0%)	155 (31.0%)	121 (24.1%)	113 (22.5%)	159 (31.8%)	253 (50.6%)
**No. children < 18 y/o**								
Average ± SD	1.16 ± 1.63	0.77 ± 1.77	0.57 ± 1.21	0.59 ± 1.10	0.75 ± 1.02	0.84 ± 1.12	0.62 ± 1.20	0.42 ± 1.21
**Education**								
< K-12	52 (10.3%)	40 (7.9%)	40 (8.0%)	28 (5.6%)	6 (1.2%)	43 (8.5%)	27 (5.4%)	15 (3.0%)
K-12 diploma	105 (20.8%)	164 (32.5%)	124 (24.8%)	118 (23.6%)	67 (13.3%)	132 (26.2%)	211 (42.2%)	139 (27.8%)
Vocational	104 (20.6%)	96 (19.0%)	81 (16.2%)	22 (4.4%)	126 (25.1%)	90 (17.9%)	40 (8.0%)	48 (9.6%)
Bachelor's degree	160 (31.7%)	130 (25.8%)	160 (32.0%)	237 (47.4%)	220 (43.8%)	126 (25.0%)	73 (14.6%)	256 (51.2%)
Master's or above	83 (16.5%)	74 (14.7%)	95 (19.0%)	95 (19.0%)	83 (16.5%)	112 (22.3%)	149 (29.8%)	42 (8.4%)
**Income**								
Much below average	100 (19.8%)	99 (19.6%)	83 (16.6%)	29 (5.8%)	48 (9.6%)	50 (9.9%)	15 (3.0%)	125 (25.0%)
Below average	107 (21.2%)	94 (18.7%)	101 (20.2%)	83 (16.6%)	89 (17.7%)	94 (18.7%)	55 (11.0%)	98 (19.6%)
Average	138 (27.4%)	176 (34.9%)	195 (39.0%)	253 (50.6%)	264 (52.6%)	239 (47.5%)	308 (61.6%)	192 (38.4%)
Above average	119 (23.6%)	105 (20.8%)	96 (19.2%)	118 (23.6%)	95 (18.9%)	99 (19.7%)	80 (16.0%)	59 (11.8%)
Much above average	39 (7.7%)	27 (5.4%)	24 (4.8%)	16 (3.2%)	6 (1.2%)	21 (4.2%)	42 (8.4%)	22 (4.4%)
**Experience with disasters**								
Yes	45 (8.9%)	67 (13.3%)	75 (15.0%)	38 (7.6%)	62 (12.4%)	54 (10.7%)	40 (8.0%)	64 (12.8%)
No	389 (77.2%)	387 (76.8%)	386 (77.2%)	415 (83.0%)	406 (80.9%)	408 (81.1%)	446 (89.2%)	372 (74.4%)
Not sure	70 (13.9%)	50 (9.9%)	39 (7.8%)	47 (9.4%)	34 (6.8%)	41 (8.2%)	14 (2.8%)	64 (12.8%)

*Maximum missing per country per variable is 4 (0.8%)*.

#### Social Norms and Sense of Communality

Participants were prompted to provide their perception of the communality in their society and its cohesion through social norms. The majority of participants tended to agree with the items comprising the Social Norm Index, with the highest agreement (55.5%) attributed to the item “Citizens follow the recommendations of authorities and emergency organizations”, and the lowest agreement (41.2%) attributed to the item “Residents in my community trust each other”. In the overall sample (*N* = 4,013), the mean of the social norm index was 3.33 ± 0.79SD (out of 5). The results suggest that social norms are perceived highest in Norway (3.66 ± 0.78SD), followed by Israel (3.57 ± 0.71SD), Italy (3.32 ± 0.77SD), Spain (3.31 ± 0.71SD), Romania (3.25 ± 0.76SD), France and Sweden (3.24 each), and lowest in Japan (3.04 ± 0.84SD). This difference is statistically significant according to One-way ANOVA (F = 33.20, *p* < 0.001).

#### Coping Skills

Participants were asked to provide their perception of their coping skills and style to adapt and manage emergencies and crises. Participants tended to widely agree with the items of the Coping Skills Index ranging from 55.9 to 61.2%. In the overall sample (*N* = 4,013), the mean of the coping skills index is 3.59 ± 0.73 (out of 5). Romanian people report the highest perception of coping skills (3.80 ± 0.63SD), followed by Spanish (3.74 ± 0.67SD) and Israelis (3.68 ± 0.57SD), while Japanese people report the lowest (3.12 ± 0.83SD) (*F* = 46.74, *p* < 0.001).

#### Public Trust

Participants were asked to indicate their levels of trust in different organizations in society. Table 2A provides the distribution of top answers to this scale (“much” and “very much”) of participants' responses across countries. The results show that the highest level of trust is assigned by the public to emergency organizations, followed by health services and civil protection agencies. The lowest level of trust is ascribed to the politicians, governments, and media.

**Table 2:** Comparison of top answers proportion for trust and perception of responsibility between countries (*N* = 4,013).

	**ISR**	**SWE**	**NOR**	**ROM**	**ESP**	**FRA**	**ITA**	**JPN**	X^2^*^^
**(A) Trust**									
The government	10.9%	28.4%	44.6%	9.0%	18.9%	18.7%	21.4%	12.0%	463.1
The civil Defense/protection	52.8%	28.2%	55.2%	19.0%	51.1%	41.3%	53.8%	13.0%	505.7
The local authority	34.9%	20.7%	43.8%	16.8%	29.7%	27.9%	26.4%	14.2%	260.2
The emergency organizations	74.4%	36.7%	62.6%	60.4%	62.3%	72.3%	75.6%	15.6%	698.5
The politicians	2.4%	14.5%	23.2%	5.6%	7.2%	10.2%	12.0%	8.6%	581.1
The media	6.8%	18.1%	24.8%	9.4%	14.4%	13.3%	15.2%	10.2%	179.5
Community organizations	41.3%	26.4%	42.2%	17.2%	32.7%	27.9%	32.0%	10.6%	273.8
Health services	33.2%	59.0%	65.2%	40.2%	67.1%	59.4%	45.2%	29.6%	404.9
**(B) Responsibility**									
The government	71.5%	67.0%	69.8%	62.8%	70.3%	58.6%	82.6%	64.4%	152.1
The civil Defense	82.9%	53.0%	55.4%	61.6%	71.6%	56.3%	84.2%	37.2%	500.1
The local authority	68.3%	61.9%	57.4%	69.0%	69.2%	51.1%	82.0%	57.0%	199.1
The health services	73.8%	69.2%	66.4%	73.4%	78.3%	63.5%	88.0%	41.6%	385.6
Your community	31.7%	27.5%	42.2%	45.4%	48.2%	31.6%	55.6%	22.8%	335.0
Yourself and your family	53.0%	47.6%	49.2%	55.4%	61.0%	39.5%	67.4%	39.6%	186.0

* *Chi-square analysis was done for all variables with all five categories of responses (degrees of freedom = 28 per item)*.

*In all analyses p-value <0.001. ISR, Israel; SWE, Sweden; NOR, Norway; ROM, Romania; ESP, Spain; FRA, France; ITA, Italy; JPN, Japan*.

The results show that except for Norway, trust in governments and politicians is extremely low across all countries. Also ranking low in the trust scale is the media. Opinions are split concerning trust in civil protection agencies, with Israel, Italy, and Norway showing relatively high levels of trust in civil protection agencies, whereas Sweden, Romania, and Japan show little trust in those agencies. Most trusted in most countries assessed are the first responders (emergency services), except for Sweden and Japan. In general, the Japanese tend to have little trust across the board.

#### Perception of Responsibility

Participants were asked to assign levels of responsibility to prepare for emergencies to different components of the society, from the government to themselves personally. Table 2B provides the distribution of top answers to this scale (“much” and “very much”) of participants' responses across countries. In the overall sample (*N* = 4,013), the results show that participants tend to assign high levels of responsibility for preparedness to all sectors, but more so when asked about the government (68.4% responded “much” or “very much”), civil protection (63.0%), local authorities (64.3%), and the health system (69.2%), and less so when asked about their community (39.4%) and themselves (51.7%).

The data shows an overall tendency to ascribe responsibility to prepare for emergencies and crises to the government and national agencies (e.g., civil protection, first responders, local authorities, and health services), and assume less personal and community responsibility for such preparedness. Italian people are the most inclined to assume personal responsibility to prepare themselves for emergencies while French individuals are the least likely to assume personal responsibility.

### Primary Outcomes

#### Emergency Preparedness

Participants were asked to indicate which items recommended for household preparedness they comply with out a list of eight items generally recommended by civil protection agencies around the globe (adapted from 17). Table 3A summarizes the compliance rates of the participants. Participants were also asked to indicate which items recommended for communal preparedness they think are complied with in their community out of a list of four items, generally recommended by civil protection agencies for communal resilience and preparedness. Table 3B summarizes the compliance rate of the participants.

**Table 3:** Distribution of compliance with (A) household adjustments and (B) communal capacities to prepare for emergencies (*N* = 4,013).

**A**	**Do you have any of the following items in place in/for your home?**	**Yes**	**No**	**Not sure/not relevant**
	A smartphone with portable charger	71.5%	21.7%	6.9%
	At least 3 liters of water per person in your family	57.0%	31.8%	11.3%
	A 4-day supply of non-perishable food items for each person in your family	62.1%	23.8%	14.1%
	A fire extinguisher	35.6%	55.3%	9.1%
	Medical needs for family members	72.9%	15.8%	11.3%
	Backup of important documents	54.7%	28.5%	16.8%
	List of vital phone numbers of family members	58.6%	30.6%	10.9%
	A household emergency plan	21.6%	61.6%	16.9%
**B**	**To the best of your knowledge, do you have any of the following items in place in your community?**	**Yes**	**No**	**Not sure/not relevant**
	Common shelters for people to be protected if need be	27.8%	45.5%	26.7%
	A community-based assistance network in which members of the community help each other during crises	27.1%	42.5%	30.4%
	Good access to emergency services during crises	42.9%	27.6%	29.5%
	A community emergency plan	24.1%	35.9%	40.0%

For each participant, the number of items indicated as complied-with for household adjustment (Table 3A) was tallied to create the Individual Preparedness Index (IPI) ranging from zero to eight. In the overall sample (*N* = 4,013), this index average at 4.44 ± 2.05SD. Table 4 provides the country-specific data of this variable. To predict IPI, a multivariate linear regression analysis was conducted. All variables found to be associated with the dependent variable (IPI) in the univariate analysis (data not shown) were introduced into the regression analysis. Analysis was done in Enter mode. The regression model is statistically significant (*F* = 68.32, *p* < 0.001) and accounts for 25.0% of the total variance of the dependent variable (see Table 5A). The results of the regression analysis suggest that adjusted to gender and age, the following variables are some of the predictors for reporting higher IPI: community preparedness (β = 0.30), Individual resilience (β = 0.17), coping skills (β = 0.129), risk awareness (β = −0.09), communication needs (β = 0.08), age (β = 0.07), and level of income (β = 0.07). Country-specific regressions analyses reveal that community preparedness is a shared predictor of IPI across all eight countries with beta values ranging from 0.277 (Spain) to 0.441 (Norway). Individual resilience is a significant predictor of IPI in Israel, Norway, Romania, Spain, France, and Japan; Age in Japan, France, Spain, and Israel; Communication needs in Japan, Spain, Romania, and Norway; Prior experience in Japan, Spain, and Italy; Coping skills in Italy, France, Israel, and Romania; Income in Spain, Italy, and Sweden; Risk awareness in Israel and France; Religiosity in Israel and Norway; Trust in Sweden and Spain; Sense of responsibility in Norway and Italy; Gender in Sweden; Number of children in Romania; and lastly, societal resilience is a significant predictor of IPI in France.

**Table 4:** Comparison of mean scores to primary outcomes between countries (*N* = 4,013).

	**ISR**	**SWE**	**NOR**	**ROM**	**ESP**	**FRA**	**ITA**	**JPN**	F^*^
Individual preparedness (IPI)^+^	3.57 (± 1.88)	3.97 (± 1.99)	5.03 (± 1.99)	4.82 (± 1.80)	5.25 (± 1.62)	4.36 (± 2.02)	5.24 (± 1.54)	3.16 (± 2.45)	82.07
Community preparedness (CPI)^++^	1.91 (± 1.26)	1.24 (± 1.16)	1.71 (± 1.36)	1.38 (± 1.31)	1.17 (± 1.19)	1.30 (± 1.31)	1.45 (± 1.40)	0.85 (± 1.27)	27.29
Individual resilience^+++^	3.68 (± 0.69)	3.62 (± 0.85)	3.58 (± 0.85)	3.53 (± 0.89)	3.80 (± 0.73)	3.60 (± 0.76)	3.69 (± 0.74)	2.83 (± 0.87)	70.17
Societal resilience^+++^	3.07 (± 0.77)	3.23 (± 0.83)	3.53 (± 0.86)	2.80 (± 0.82)	3.04 (± 0.79)	3.08 (± 0.84)	3.17 (± 0.82)	2.84 (± 0.82)	38.83

* *One-way ANOVA's F. All values are significant at a p-value < 0.001*.

+ *Scale ranges between 0 and 8*.

++ *Scale ranges between 0 and 4*.

+++ *Scale ranges between 1 and 5*.

*ISR, Israel; SWE, Sweden; NOR, Norway; ROM, Romania; ESP, Spain; FRA, France; ITA, Italy; JPN, Japan*.

**Table 5:** Result of linear regression analysis to predict individual resilience (*N* = 4,013).

	**Unstandardized coefficients**		**Standardized coefficients**	***t***	**Sig**.
	**B**	**Std. error**	**Beta**		
**(A) MODEL 1 – PREDICTION OF INDIVIDUAL PREPAREDNESS INDEX (IPI)**					
(Constant)	0.290	0.300		0.968	0.333
Gender	0.018	0.061	0.005	0.300	0.764
Age	0.011	0.002	0.069	4.435	0.000
Religiosity	−0.005	0.049	−0.002	−0.104	0.917
Children	−0.118	0.048	−0.038	−2.463	0.014
Education	0.030	0.062	0.007	0.485	0.628
Income	0.130	0.030	0.066	4.321	0.000
Social Norms and Communality	−0.111	0.049	−0.044	−2.252	0.024
Coping Skills	0.363	0.057	0.129	6.377	0.000
Individual resilience	0.414	0.051	0.170	8.076	0.000
Community Preparedness	0.470	0.026	0.303	18.293	0.000
Trust	−0.041	0.060	−0.016	−0.684	0.494
Responsibility	0.079	0.045	0.032	1.752	0.080
Societal Resilience	−0.101	0.055	−0.042	−1.818	0.069
Communication Needs	0.231	0.053	0.082	4.35	0.000
Digital Literacy	0.062	0.039	0.029	1.583	0.114
Risk Awareness	−0.040	0.007	−0.087	−5.803	0.000
**(B) MODEL 2 – PREDICTION OF INDIVIDUAL RESILIENCE**					
(Constant)	−0.332	0.076		−4.356	0.000
Gender	−0.013	0.020	−0.008	−0.637	0.524
Age	0.002	0.001	0.037	3.053	0.002
Education	−0.049	0.020	−0.029	−2.443	0.015
Income	0.018	0.010	0.022	1.816	0.069
Social Norms and Communality	0.011	0.016	0.011	0.703	0.482
Coping Skills	0.470	0.017	0.407	27.914	0.000
Individual Preparedness	0.044	0.005	0.108	8.115	0.000
Community Preparedness	0.007	0.009	0.011	0.818	0.413
Trust	0.024	0.020	0.022	1.217	0.224
Responsibility	0.122	0.014	0.121	8.436	0.000
Societal Resilience	0.064	0.018	0.065	3.568	0.000
Communication Needs	0.042	0.017	0.037	2.445	0.015
Digital Literacy	0.199	0.012	0.227	16.350	0.000
Risk Awareness	0.016	0.002	0.087	7.333	0.000

#### Resilience

Table 4 provides the breakdown of the differences in resilience between the studied countries. In all countries, except Japan, individual resilience is ranked higher than societal resilience. In line with the individual preparedness index, individual resilience is highest among Spanish, Italian, Israeli, Swedish, and French people. Individual resilience is lowest among Japanese respondents. In contrast to individual resilience, societal resilience is reported highest in Norway and Sweden and lowest in Romania and Japan.

**Figure 2:**
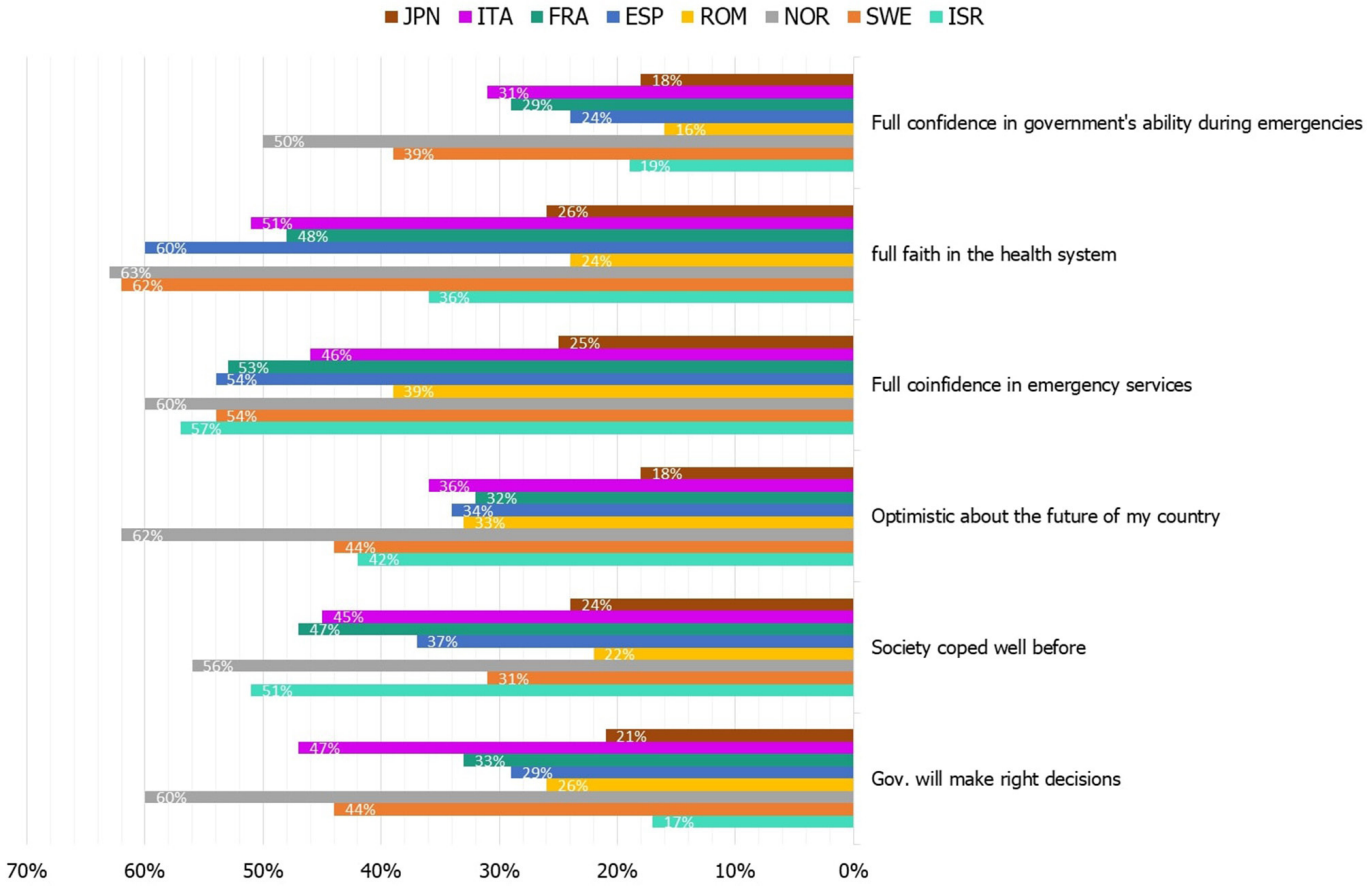
Country comparison of the distribution of agreement (“agree” and “strongly agree”) with items comprising the Societal Resilience Index (*N* = 4,013). ISR, Israel; SWE, Sweden; NOR, Norway; ROM, Romania; ESP, Spain; FRA, France; ITA, Italy; JPN, Japan; Gov., Government.

A comparison of the components comprising the societal resilience index revealed differences between the countries. For instance, confidence in the government's ability to take care of all aspects relevant to overcoming crises ranges from 16% of top answers (“agree” and “strongly agree”) (Romania) to 50% (Norway). Trust in the health services to care for the population in crisis ranges from 24% (Romania) to 63% (Norway). Confidence in emergency services to protect the country's population ranges from 25% (Japan) to 60% (Norway). Japanese are the least optimistic about the future of their country (18%), as opposed to Norwegians who far lead other countries with 62%. While Israelis have the smallest confidence in their government making the right decisions (17%), the country ranks second in the perception of societies coping with past crises (51%). Romanian and Japanese people tend to rank all components relatively low. In most countries, emergency services and the health systems enjoy high levels of confidence (compared to governments). See complete details in Figure 2.

To predict individual resilience, a multivariate linear regression analysis was conducted. All variables found to be associated with the dependent variable (Individual Resilience) in the univariate analysis (data not shown) were introduced into the regression analysis. The regression model is statistically significant (*F* = 277.43, *p* < 0.001) and accounts for 52.6% of the total variance of the dependent variable (see Table 5B). The results of the regression analysis suggest that adjusted to gender and age, the following variables are predictors for reporting higher individual resilience: coping skills (β = 0.41), digital literacy (β = 0.23), sense of responsibility (β = 0.12), individual preparedness (β = 0.11), risk awareness (β = 0.09), societal resilience (β = 0.07), age (β = 0.04), communication needs (β = 0.04), and level of education (β = −0.03). Country-specific regressions analyses reveal that coping skills and responsibility are shared predictors of individual resilience across all eight countries. Individual resilience is further predicted by religiosity in Sweden, the number of children in Norway, social norms and communality in Romania, level of education in Japan, Income in Italy, Japan, and Spain, and risk awareness in Japan, France, Spain, Romania, and Norway.

Societal resilience is another primary outcome of this study. To assess this construct, participants were asked to provide their answers to eight items measuring their perception of their community and society to adapt and adjust in the face of hardship. Table 6 summarizes the distribution of responses to the items in this scale. For each participant, the societal resilience index was computed as the mean score of their responses to these eight items.

**Table 6:** Distribution of responses to societal resilience index items (*N* = 4,013).

**To what extent do you agree with each of the following statements relating to your country in the context of emergency preparedness**	**Disagree**	**Neither**	**Agree**
My government will make the right decision during a time of crisis.	33.0%	32.2%	34.7%
I have full confidence in the ability of the emergency services of my country to protect our population.	19.9%	31.6%	48.5%
My society has coped well with past crises.	21.3%	39.5%	39.2%
I am optimistic about the future of my country.	29.1%	33.3%	37.6%
In my society, there is a high level of social solidarity (mutual assistance and concern for one another).	24.2%	37.0%	38.8%
In my society, there is a reasonable level of social justice.	29.3%	37.8%	32.9%
I have full faith in the ability of my country's health system to care for the population in crisis.	21.1%	32.5%	46.4%
I have complete confidence in the ability of my government to take care of all aspects relevant to overcoming crises.	39.8%	32.0%	28.2%

To predict societal resilience, a multivariate linear regression analysis was conducted. All variables found to be associated with the dependent variable in the univariate analysis (data not shown) were introduced into the regression analysis. The regression model is statistically significant (*F* = 349.78, *p* < 0.001) and accounts for 60.1% of the total variance of the dependent variable. The results of the regression analysis suggest that adjusted to gender and age, the following variables are predictors for reporting higher societal resilience: trust (β = 0.59), social norms and communality (β = 0.20), communication needs (β = 0.09), individual resilience (β = 0.05), individual preparedness (β = 0.04), risk awareness (β = 0.04), and age (β = 0.03).

## Discussion

The results of this study suggest that all hypotheses can be accepted. Findings show that Individual resilience and preparedness (IPI), as well as societal resilience, are all associated with communality, coping skills, trust, and assuming personal responsibility to prepare for emergencies. Correlations were reported in the directions hypothesized. In this regard, the current study resonates with the findings of prior research that showed that increased resilience and readiness is associated with the perception of responsibility (33), coping skills (34), levels of trust in local and national entities (35), and other contextual and target factors (21). The current study provides evidence that the abovementioned correlations can be considered universal and relevant to people of different backgrounds and nationalities.

The primary outcome of this study, namely societal resilience, has been found to correlate with numerous other factors. These include some contextual factors, such as level of household income and religiosity. Previous studies have identified an association between the level of income, socio-economic characteristics, and religiosity with emergency preparedness and resilience (42, 43). These socio-economic contexts are related to both levels of education of the population at large, as well as that of the formal responders, and to the funds that may be allocated to build a robust risk reduction program, thus contributing to societal resilience (44).

More importantly, many target factors that can be modified and changed through policies, such as a sense of communality, trust in societal entities, risk awareness, coping skills, and sense of responsibility, are associated with societal resilience. Risk awareness and trust contribute to the participation of the populace in disaster risk reduction activities and thus enhance mitigation strategies and a more effective risk management program (44, 45). Furthermore, it has been claimed that trust is intrinsic to the development of societal resilience, dependent on the full scale of confidence at both the local, state, and multi-national level, such as the overall EU-level, concerning European countries (46).

Several elements were found to be of high contribution to predicting resilience. Coping skills and perceived responsibility to prepare for adversities were identified as having the highest contribution to predicting individual resilience, while the trust of the public in the varied authorities and social norms were found to be of higher contribution to predicting societal resilience. Perceived responsibility and trust in authorities are target factors. As such, they can more easily be enhanced in the respective countries, dependent on efforts being invested to increase the skills and competencies of the civil societies, transparency in policy and decision-making, and the involvement of the public as an important partner in managing the adversity (45, 47). In contrast, constructs such as social norms represent contextual factors that are much more complex to modify. Nonetheless, solutions that aim to impact them too are vital, in order to increase both individual and societal resilience.

In line with previous studies (48, 49), as far as trust goes, emergency services and the health system are usually enjoying a high level of public trust (58 and 49% top answers, respectively). In contrast, politicians and the media are ranked lowest on the trust scale with 66 and 56% (respectively) indicating the top-bottom options for these entities (49, 50). It is important to note variations between countries. Israeli, Japanese, and Romanian populations reported the lowest levels of trust in their respective governments, while Norway reported a much higher level of such trust. One characteristic that may contribute to this diversity is the collectivism vs. individualism approach to leadership and decision-making (44, 51, 52). The first three countries' management systems are more frequently based on the individualistic approach, involving fewer officials and stakeholders while designing their policies. In contrast, Norway's governance system is characterized by a more collaborative leadership, striving to maintain transparency in policy-making and the involvement of diverse stakeholders in the process (53).

Another notable diversity is the different perceived trust in civil defense/protection agencies. For example, Romanian, Swedish and Japanese people report lower trust levels in their civil protection/defense agencies compared to the other investigated countries. This is most probably derived from the historical legacies of the different countries. Japan is known for its post-WWII pacifist constitution (54); Romania is amid an ongoing instability concerning imperial claims due to the struggle between “East and West” (55, 56); and the Swedish Armed Forces are perceived by some as having a credibility gap, due to low transparency and lack of communication with the public (57).

Trust is a major component in creating the infrastructure on which societal resilience can be established. Previous studies show that public trust in government and emergency services is key in supporting resilience growth in those societies (58, 59). Trust is a major driver in public compliance with regulations, as demonstrated with the case of COVID-19, for example (31, 60). Trust is also likely to allow recruitment of the public by emergency services as a partner to facilitate a more optimized response to crises.

In terms of responsibility, the data shows that overall participants from different societies tend to project responsibility to prepare for emergencies on the government and national authorities (68%) and assume less of it personally (52%). This finding is similar to that reported in other studies looking into the perception of responsibility (19, 33). Findings from these and other studies show that having a heightened sense of personal responsibility to prepare for emergencies is an important component in driving households' preparedness and consequently more communal resilience (61).

It may also be conjectured that experiencing substantial adversities may negatively impact the perceived trust of the varied populations concerning their governing systems, confidence in their ability to provide aid, and belief in their capacity for decision-making. Three examples of such perceptions can be seen concerning Japan, Israel, and Romania. The trust of the Japanese populace in their government's management of adversities considerably decreased following the 2011 earthquake and Fukushima's radiological spill (62, 63). Lower levels of confidence in governmental decision-making have been reported following the security and terror events in Israel (64, 65). Romania is located at the margin of Europe and is thus more exposed to geopolitical risks and it is also “one of the most seismically active countries in Europe” [(66), p. 667]. Conversely, countries such as Norway that relatively had not experienced as many adverse events in the past decade (apart from the Utoya terror attack), are characterized by higher levels of trust and confidence in their governance system (67).

This study suggests that both contextual and target factors should be considered when approaching discussion of societal resilience. Some factors are more relevant as candidates for policy change, namely the target factors. Policy maker may want to consider risk communication that is more focused toward empowerment of the public to assume personal responsibility and foster self-efficacy. Other efforts should be done to re-establish trust between the public and sectors that do not enjoy high levels of trust with the public, such as the political level. In parallel, it is important for planners to address the specific contextual characteristics that exist for each society (and even locality) to ensure that efforts are tailored to the properties of each society.

### Limitations

This study has some limitations. First, technical constraints limited the national samples sizes to 500 in each country. While in some countries this sample size is adequate to provide a representative sample of the entire population, in other participating countries it may be difficult to fully cover all different groups in the society. Second, this study was performed online. Accessing participants through online channels proves to be a very rapid way of collecting responses in wide geographical distribution. Nonetheless, it limits the conclusions to participants with the minimal set of skills needs to perform the questionnaire online. Therefore, findings should be limited to individuals with adequate digital literacy and access to digital tools. Third, as is the case with other cross-sectional studies, this study assessed attitudes and opinions at a certain point in time. Fluctuations in circumstances surrounding the study could register a temporal effect on individuals' perceptions. Fourth, some aspects included in the questionnaire may be prone to social desirability bias. For example, questions pertaining to local coping capacities trust, and personal preparedness could be skewed due to participants' will to make an impression on the survey planners. This bias was reduced to a minimum by the text explaining to the participants that all information collected is anonymous.

## Conclusions

The results of this study show that there are commonalities and differences between societies across Europe and outside concerning societal resilience and emergency preparedness. In particular, this study suggests that societies share a model to explain and predict resilience and readiness, which is relevant regardless of the nationality of participants. Nevertheless, when zooming into each society, differences can be found in attitudinal factors associated with said resilience. Essentially, the conclusion of this study, in this regard, is that while there is a common model to promote resilience, different societies have different attributes that either place them high or low on the societal resilience scale.

Importantly, the findings suggest that societies have little trust in governments and varied levels of trust toward emergency services, health services, and other stakeholders relevant for disasters and emergencies. Trust can be fostered through appropriate risk communication initiatives that value transparency, accuracy, simplicity, and timing. Since trust is a major component in societal resilience and is even found in this study to serve as a predictor of societal resilience, it is imperative that wherever trust between the public and the authorities is not strong enough, it will be strengthened.

Lastly, the findings of this study suggest that while common models for societal resilience may be presented on a pan-human basis, specific variations that are cultural dependent can emerge. Future research could focus on explaining socio-cultural variations in societal resilience across societies and propose additional similarities and differences in the factors contributing to societal resilience.

## Data Availability Statement

The raw data supporting the conclusions of this article will be made available by the authors, without undue reservation.

## Ethics Statement

The studies involving human participants were reviewed and approved by the Ethical Committee of the Tel-Aviv University (approval number 0002377-1 dated November 25, 2020). In addition, this study was approved by the Ethical Committee of the Norwegian Research Council. Subsequently to this approval, the study was granted exemption from further approvals in each participating country. The patients/participants provided their written informed consent to participate in this study.

## Author Contributions

MB, KP, NS, and BA contributed to the conception and design of the study. MB organized the database, performed the statistical analysis, and wrote the first draft of the manuscript. BA wrote sections of the manuscript. All authors contributed to manuscript revision, read, and approved the submitted version.

## Funding

The research leading to these results has received funding from Horizon 2020, the European Union's Framework Programme for Research and Innovation (H2020/2014-2020) under Grant Agreement No. 882850.

## Conflict of Interest

The authors declare that the research was conducted in the absence of any commercial or financial relationships that could be construed as a potential conflict of interest.

## Publisher's Note

All claims expressed in this article are solely those of the authors and do not necessarily represent those of their affiliated organizations, or those of the publisher, the editors and the reviewers. Any product that may be evaluated in this article, or claim that may be made by its manufacturer, is not guaranteed or endorsed by the publisher.
